# Interval between Intra-Arterial Infusion Chemotherapy and Surgery for Locally Advanced Oral Squamous Cell Carcinoma: Impacts on Effectiveness of Chemotherapy and on Overall Survival

**DOI:** 10.1155/2014/568145

**Published:** 2014-05-18

**Authors:** Chih-Fung Wu, Chien-Hsing Lee, Edward Hsi, Chung-Ho Chen, Jen-Yang Tang

**Affiliations:** ^1^Department of Surgery, Faculty of Medicine, College of Medicine, Kaohsiung Medical University Hospital, Kaohsiung 807, Taiwan; ^2^Department of Nursing, Min-Hwei Junior College of Health Care Management, Tainan 736, Taiwan; ^3^Department of Medical Research, Kaohsiung Medical University Hospital, Kaohsiung 807, Taiwan; ^4^Cancer Center, Kaohsiung Medical University Hospital, Kaohsiung 807, Taiwan; ^5^Department of Oral Maxillofacial Surgery, Kaohsiung Medical University Hospital, Kaohsiung 807, Taiwan; ^6^Department of Radiation Oncology, Faculty of Medicine, College of Medicine, Kaohsiung Medical University, Kaohsiung 807, Taiwan; ^7^Department of Radiation Oncology, Kaohsiung Medical University Hospital, Kaohsiung 807, Taiwan; ^8^Department of Radiation Oncology, Kaohsiung Municipal Ta-Tung Hospital, Kaohsiung 801, Taiwan

## Abstract

*Background.* The interval between intra-arterial infusion chemotherapy (IAIC) and surgery was investigated in terms of its effects on survival in patients with locally advanced oral squamous cell carcinoma (OSCC). *Methods.* This retrospective study analyzed 126 patients who had completed treatment modalities for stage IV OSCC. All patients were followed up for 3 years. Kaplan-Meier and Cox regression methods were used to determine how survival was affected by general factors, primary tumor volume, TNM stage, and duration of neoadjuvant chemotherapy. *Results.* In 126 patients treated for locally advanced OSCC by preoperative induction IAIC using methotrexate, multivariate analysis of relevant prognostic factors showed that an IAIC duration longer than 90 days was significantly associated with poor prognosis (hazard ratio, 1.77; *P* = 0.0259). *Conclusions.* Duration of IAIC is a critical factor in the effectiveness of multimodal treatment for locally advanced OSCC. Limiting the induction course to 90 days improves overall survival.

## 1. Introduction

Invasive oral squamous cell carcinoma (OSCC) accounts for most malignant disorders of the oral cavity. In Taiwan, oral cancer is currently the fourth most common cancer in males [1]. Although 80–90% of patients with early stage OSCC are cured, outcomes remain poor in those with advanced stage tumors [2, 3]. In OSCC stages IVA and IVB, short-induction chemotherapy achieves high primary remission rate with possible organ preservation and acceptable toxicity [4]. For controlling the progression of cancer, intravenous systemic or regional intra-arterial infusion chemotherapy (IAIC) via superselective catheterization has varying success [5–7]. Kaohsiung Medical University Hospital (KMUH) and many other centers have used IAIC for decades to treat head and neck cancers, and preoperative superselective infusion generally obtains a good response with minimal toxicity [8–13]. For patients in early stages of OSCC, the main curative modality is either surgery or radiotherapy [14]. For patients in advanced stages, however, a multidisciplinary approach is usually applied; for example, dissection of the primary tumor and selected regional lymph nodes is combined with salvage treatments such as radiotherapy and chemotherapy, which have a high therapeutic ratio [14, 15]. For OSCC in an advanced stage but still operable, the typical treatment modality is radical ablative surgery followed by radiotherapy or radiochemotherapy, which is the same treatment applied in laryngeal and pharyngeal squamous cell carcinoma [16, 17].

However, no clinical trials have compared the beneficial effects between IAIC and surgery. At our hospital, systemic intravenous chemotherapy is not the typical neoadjuvant therapy for OSCC. The standard therapy is IAIC followed by surgical intervention and postoperative concurrent chemoradiotherapy. Therefore, this retrospective study investigated how the IAIC-surgery interval affects outcomes of treatment for locally advanced OSCC.

## 2. Materials

### 2.1. Patient Characteristics

This study retrospectively analyzed 126 consecutive patients who had received IAIC treatment for OSCC at KMUH from 2005 to 2010. Inclusion criteria included histologically confirmed primary OSCC originating from cheek mucosa, no distant metastasis at presentation, and no previous treatment of a malignancy at any oral cavity site. The analysis excluded patients who did not complete the therapeutic protocol and those who had incomplete medical records. The remaining patients who had locally advanced IVA or IVB lesions (without distant metastasis) and who met the enrollment criteria were enrolled for further analysis. Patients who had received a multidisciplinary therapeutic treatment regimen of regional chemotherapy, surgery, and irradiation were retrospectively analyzed. Institutional review board approval was obtained before beginning data collection. Due to the small number of female patients, only male patients were enrolled. The 126 enrolled patients had a median age of 50.6 years. The AJCC TNM staging system was applied retrospectively in all patients (Table 1). The mean follow-up period was 29.2 months (range 4.6–84.8 months). The date of the last follow-up was recorded as the date of the last admission, the date of the last outpatient visit, or the date of death.

### 2.2. Pretreatment Evaluation

The pretreatment evaluation included history, clinical examination, hematological evaluation, and primary tumor biopsy. Before beginning treatment, the extent of the spread was determined by bone scan, chest X-ray, contrast-enhanced computed tomography (CT) of the head and neck, and ultrasound scan of the abdomen. All patients were restaged according to 7th edition of the AJCC cancer-staging manual for the oral cavity.

### 2.3. Treatment Protocol

A totally implantable port-catheter system (Jet Port Plus Allround; PFM, Cologne, Germany) was used for continuous IAIC as described previously [11]. Briefly, the catheter tip was placed on a branch of the feeding artery proximal to the tumor. The tumorous area and the proper position of the catheter tip were confirmed by staining with patent blue V (Guerbet Co, France).

Methotrexate 50 mg was infused intra-arterially every 24 hrs with leucovorin 6 mg given intramuscularly every 6 hrs during the course of methotrexate infusion. Methotrexate was given continuously for a mean period of 7.5 days and the regimen was changed to weekly bolus of 25 mg via intra-arterial route. The patients received IAIC for 2-3 months before surgery.

No patients had received other treatments before undergoing IAIC. Surgical resection including the primary site with neck dissection was performed 2 weeks after completion of intra-arterial chemotherapy when the mucositis had subsided. After surgical resection, OSCC risk factors identified by pathology were evaluated before performing radiotherapy.

### 2.4. Treatment Evaluation

Each patient was clinically evaluated in follow-up examinations performed at 1- to 3-month intervals. Disease-free survival was defined as the date of treatment to the date that evidence of recurrence was noted.

### 2.5. Statistical Analysis

The association between IAIC duration and treatment response was analyzed by Pearson chi-square test. Survival rates were calculated by Kaplan-Meier method and compared by Cochran-Mantel-Haenszel test. Overall survival (OS) was calculated from the time of initial diagnosis to the time of death of any cause. Patients who had survived or who were disease-free at the end of the follow-up were censored. Potential confounding factors were adjusted and analyzed by Cox proportional hazard regression analysis. The statistical analysis was performed with JMP version 9. All statistical tests were performed at a 0.05 significance level. Estimated results were reported with the hazard ratio (HR) and 95% confidence interval (CI).

## 3. Results

### 3.1. Characteristics of Prognostic Factors

In this retrospective review of Taiwan population of consecutive patients who had received IAIC for OSCC, the inclusion criteria were the following: histologically confirmed primary OSCC originating from cheek mucosa, no distant metastasis at presentation, and no history of treatment for a malignancy at an oral cavity site. Patients were excluded if they did not complete the therapeutic protocol or if their medical records did not contain the data required for the analysis in this study. After the exclusions, 126 patients who had locally advanced lesions in OSCC stages IVA or IVB (without distant metastasis) and who met all enrollment requirements were enrolled for further analysis (Table 1). The sites of locally advanced OSCC in the enrolled patients included the tongue (15.9%), gums (21.4%), buccal mucosa (54%), and other (8.7%) (Table 2).

### 3.2. Contribution of Prognostic Characteristics to Mortality in Locally Advanced Buccal Cancer

Since the review of medical records for the enrolled patients revealed varying durations of IAIC, this study determined the optimal duration of IAIC in terms of overall survival. A receiver operating characteristic (ROC) curve analysis of the sensitivity and specificity of various IAIC durations in predicting 3-year overall survival in the 126 patients with locally advanced OSCC indicated that the optimal duration of IAIC was 90 days. Univariate Cox regression analysis indicated that longer durations of IAIC (>90 days) (hazard ratio [HR], 1.95; 95% confidence interval [CI] 1.19–3.24; *P* < 0.0085), mucosa sites compared to gum sites (HR, 2.27; 95% CI, 1.11–5.25; *P* = 0.0235), tongue sites compared to gum sites (HR, 2.50; 95% CI, 1.01–6.47; *P* = 0.0477), and other sites comparing to gum sites (HR, 2.93; 95% CI, 1.07–7.96; *P* = 0.0360) were each associated with OS in patients with OSCC (Table 3).

**Figure 1:**
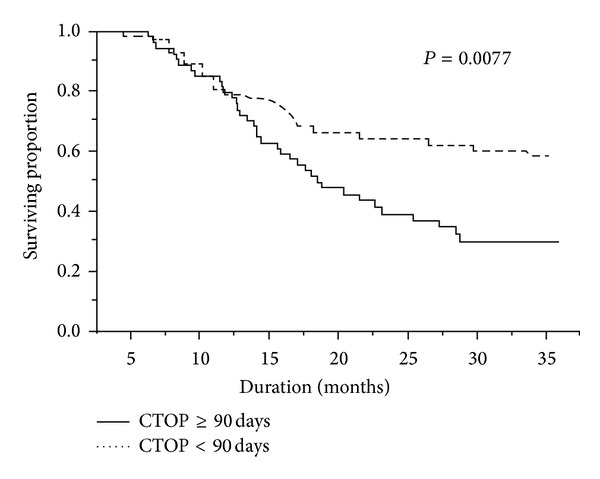
Interval between intra-arterial infusion chemotherapy (IAIC) and surgical resection: effect on overall survival. Kaplan-Meier plots of overall survival in patients with locally advanced oral squamous cell carcinoma: IAIC-surgery intervals ≥90 days versus IAIC-surgery intervals <90 days. CTOP: duration of induction chemotherapy before surgical resection.

Age and differentiation were not significantly associated with survival. Multivariate analysis was used to assess the effect of long duration of IAIC (>90 days) on OS, independently of other factors. The results showed that >90 days IAIC retained a statistically significant association with OS (HR, 1.77; 95% CI, 1.07–2.97; *P* = 0.0259). According to the multivariate analysis, mucosa comparing to gum was the only other significant risk factor with regard to OS (HR, 2.08; 95% CI, 1.02–4.84; *P* = 0.0451). Kaplan-Meier survival curves showed that prolonged (>90 days) neoadjuvant chemotherapy (Figure 1) was associated with poor survival (Log-Rank *P* = 0.0077).

## 4. Discussion

After excluding patients with early stage OSCC, female patients, and patients with incomplete medical records, the final analysis included 126 patients registered at KMU Hospital for treatment of locally advanced OSCC. Multivariate analysis of relevant prognostic factors showed that a long duration of IAIC was significantly associated with a poor prognosis. The maximum benefit of IAIC was observed when IAIC was limited to 90 days. To the best of our knowledge, this study is the first to report survival rates and prognostic factors in patients who have received induction IAIC for locally advanced OSCC.

The use of IAIC to treat locally advanced cancers began in the 1980s [13]. Since then, it has been widely used as a palliative modality to treat various conditions, particularly head and neck cancer. Compared to intravenous systemic chemotherapy, the theoretical advantage of IAIC is its capability to deliver higher drug concentrations to the tumor site but with lower systemic toxicity [18]. However, no guidelines have been established for selecting the optimal duration of induction chemotherapy before surgical resection (CTOP) for locally advanced OSCC, and no guidelines are available for predicting how the duration of induction chemotherapy affects the oncologic outcome. Our study showed that the duration of IAIC correlated with overall survival, and the ROC assay showed that patients who had received 90 days of IAIC presented with the best survival. However, Kaplan-Meier survival analysis showed that hazard ratios varied in a time-dependent fashion (Figure 1). The IAIC revealed a protective effect in the early stage of a multiple treatment modality and apparently improved survival (Figure 1, dotted line). However, treatment outcomes worsened as the duration of chemotherapy increased and as the delay in surgical intervention increased. The probable explanation is that prolonged IAIC causes selective killing of certain strains of malignant cells and causes fibrosis of locoregional tissue. Indicators of a poor outcome of subsequent salvage treatment, radiotherapy, or adjuvant chemotherapy include the presence of a radiotherapy-resistant strain and a hypoxic environment.

The Cox regression assay of age, differentiation, and CTOP duration revealed one statistically significant factor. The Cox regression results were consistent with the hypothesis based on chi-square analysis. Despite recent advances in aggressive combined treatment regimens, for example, radical surgery, chemoradiation, neoadjuvant chemotherapy, and target therapy, the long-term survival of OSCC patients has not substantially improved and responses to different treatment modalities are still difficult to predict. However, high morbidity and mortality rates are expected in patients who present with an advanced stage of the disease, low Karnofsky status, or bad habits [19]. Despite advances in the diagnosis and therapeutic treatment of OSCC, the prognosis for advanced stages of the disease remains poor.

## 5. Conclusion

This study revealed that when followed by appropriate surgical resection, selective lymph node dissection, and adjuvant radiotherapy, IAIC has an important contributing role in improving survival of OSCC by reducing systemic toxicities. However, the maximal benefit of IAIC is conferred when chemotherapy is limited to 90 days. Prolonged neoadjuvant IAIC obtains adverse outcomes.

## Figures and Tables

**Table 1:** Patients' characteristics.

Characteristics	Number of patients who completed treatment (%)	Number of all patients (%)
Number of patients	126	1,459
Median age (years)	50.6	52.5
Age range (years)	28–82	22–87
Differentiation		
I	74 (72.5)	760 (64.0)
II	26 (25.5)	394 (33.2)
III	2 (2)	33 (2.7)
T stage		
1	1 (0.8)	341 (23.4)
2	12 (9.5)	420 (28.8)
3	8 (6.3)	122 (8.4)
4	1 (0.8)	3 (0.2)
4A	104 (82.5)	563 (38.6)
4B	0 (0)	8 (0.5)
N stage		
0	21 (16.7)	633 (43.4)
1	55 (43.7)	505 (34.6)
2	49 (38.9)	308 (21.1)
3	1 (0.8)	11 (0.8)
IAIC-OP interval		
Range (months)	0.4–14	0–14.8
≥90 days	54 (42.9)	132 (45.2)
<90 days	72 (57.1)	160 (54.8)

**Table 2:** Tumor distribution in the oral cavity.

Tumor site	*N* (%)
Buccal mucosa	68 (54)
Gum	27 (21.4)
Tongue	20 (15.9)
Other	11 (8.7)

Total	126 (100)

**Table 3:** Univariate and multivariate analysis of prognostic factors in locally advanced OSCC receiving neoadjuvant IAIC.

	Survival					
	Univariate			Multivariate		
	HR	95% CI	*P* value	HR	95% CI	*P* value
Age	0.98	0.95–1.00	0.0890			
Differentiation						
I	1.00					
II + III	1.78	0.97–3.14	0.0616			
Sites						
Gum	1.00			1.00		
Mucosa	2.27	1.11–5.25	0.0235	2.08	1.02–4.84	0.0451
Tongue	2.50	1.01–6.47	0.0477	2.24	0.90–5.83	0.0826
Others	2.93	1.07–7.96	0.0360	2.41	0.88–6.66	0.0865
IAIC-OP interval ≥90	1.95	1.19–3.24	0.0085	1.77	1.07–2.97	0.0259
